# Environmental Drivers and Explainable Modeling to Resolve Trace Metal Dynamics in a Lotic System

**DOI:** 10.3390/toxics14030215

**Published:** 2026-02-28

**Authors:** Akasya Topçu, Dilara Gerdan Koç, İlknur Meriç Turgut, Serkan Taşdemir

**Affiliations:** 1 Department of Fisheries and Aquaculture Engineering, Faculty of Agriculture, Ankara University, 06110 Ankara, Türkiye; atopcu@ankara.edu.tr; 2 Department of Agricultural Machinery and Technologies Engineering, Graduate School of Applied and Natural Sciences, Ankara University, 06110 Ankara, Türkiye; dgerdan@ankara.edu.tr; 3 Department of Fisheries and Aquaculture Engineering, Graduate School of Applied and Natural Sciences, Ankara University, 06110 Ankara, Türkiye; serkantasdemir@gmail.com

**Keywords:** riverine pollution dynamics, trace metals, temporal variability, machine learning, ensemble modeling

## Abstract

Trace metal contamination in lotic freshwater systems exhibits pronounced heterogeneity arising from coupled hydrological connectivity, geochemical partitioning, and anthropogenic forcing, complicating exposure characterization in urban and peri-urban catchments. Addressing this complexity requires integrative analytical approaches capable of deciphering system-level controls, prompting an investigation of the environmental structuring and governing controls of dissolved trace metal signatures in a human-impacted stream using a system-oriented computational framework. To capture temporal variability associated with seasonal hydrological contrasts and heterogeneous pollution inputs, a station-based, season-resolved sampling strategy was implemented during the wet and dry seasons. Physicochemical gradients (pH, temperature, dissolved oxygen, and electrical conductivity), inorganic nitrogen species (NH_3_, NO_2_^−^, and NO_3_^−^), and phosphorus fractions (total phosphorus, TP; total orthophosphate, TOP; soluble reactive P, SRP) were jointly analyzed with dissolved concentrations of chromium (Cr), copper (Cu), nickel (Ni), lead (Pb), cadmium (Cd), mercury (Hg), and arsenic (As). Regression-based machine learning models were used to quantify element-specific sensitivities to hydrochemical drivers under wet–dry periods and to identify optimal predictive configurations. Predictive performance was consistently high for trace metals (*R*^2^ generally >0.95), with Random Forest providing the best accuracy for Cr, Ni, Pb, Cd, As, and Hg, whereas Cu was most reliably captured by an XGBoost tree ensemble (*R*^2^ = 0.994). Explainability analyses revealed heterogeneous, metal-specific control regimes: Cr was primarily driven by temperature, Ni by NO_2_^−^ and redox-sensitive conditions, Cd by NH_3_ and temperature, and As by Hg in combination with phosphorus-related and redox-linked proxies, while Pb showed comparatively lower predictability relative to other metals. Trace metal distributions are therefore structured primarily by differential environmental sensitivity rather than uniform source-driven inputs, reinforcing the need for integrative computational frameworks when interpreting freshwater contamination under intensifying anthropogenic and climatic pressures.

## 1. Introduction

Within the framework of sustainable development, maintaining freshwater quality has emerged as a critical challenge as intensifying anthropogenic pressures increasingly compromise aquatic ecosystems. The Intergovernmental Panel on Climate Change emphasizes that environmental sustainability requires integrating environmental protection with socio-economic development while explicitly accounting for climate-change impacts [1]. Sustainability assessments commonly rely on indicators of resource efficiency, pollution control, and ecosystem conservation [2]; these metrics notwithstanding, freshwater systems remain disproportionately vulnerable, with nearly 79% of surface water resources in Türkiye reported as polluted [3].

This vulnerability is strongly shaped by chronic chemical stressors, particularly trace metals, which exert a persistent and ecotoxicologically consequential pressure on freshwater environments. Sustained inputs derived from industrial, agricultural, domestic, and mining activities compromise water quality, restrict the availability of usable freshwater resources, and perturb ecological processes, with industrial effluents operating as dominant point sources that underpin long-term metal enrichment and attendant ecological and human health risks [4]. Across stream ecosystems, the distribution of trace metals therefore remains a focal concern in environmental toxicology, as concentrations exhibit pronounced spatial and temporal variability governed by the coupled action of hydrological transport, sedimentary processes, and water chemistry. Early transport studies demonstrated that even in relatively simple river systems, metals such as Cu and Zn exhibit downstream concentration gradients governed by flow regime and sediment interaction [5]. Subsequent work expanded this understanding by showing that advection–dispersion processes alone are insufficient to explain observed patterns unless coupled with physicochemical controls such as pH, redox conditions, and suspended solids, which regulate metal partitioning and mobility [6,7]. These insights established the mechanistic foundation upon which contemporary predictive modeling of metal fate in lotic environments is built.

Recent research on inland waters further demonstrates that metal contamination must be evaluated within an integrated water–sediment framework rather than solely through dissolved-phase measurements. Nodefarahani et al. [8] reported spatially differentiated metal enrichment in surface sediments of Namak Lake (Iran), underscoring the regulatory role of sediments in governing metal mobility and environmental risk.

As monitoring datasets increased in spatial coverage and temporal resolution, computational approaches diversified into three dominant paradigms: physically based models, machine-learning (ML) models, and hybrid frameworks integrating both. Physically based models rely on conservation equations to resolve mass transport and have been widely applied to simulate trace metal dynamics under varying hydraulic conditions [9,10]. Their predictive skill improves when environmental covariates regulating metal speciation—particularly temperature, pH, dissolved oxygen, and electrical conductivity—are incorporated into advection–diffusion formulations, underscoring the tight coupling between hydrodynamics and water chemistry [11]. Nevertheless, several studies have highlighted that incomplete representation of in-channel attenuation, hyporheic exchange, and sediment–water interactions can lead to systematic concentration overestimation, particularly during low-flow periods [7,12].

In parallel with mechanistic modeling, data-driven approaches have gained prominence as an alternative means of capturing the nonlinear relationships that characterize real-world river systems influenced by heterogeneous pollution sources. Artificial neural networks and random forest (RF) algorithms are now among the most frequently applied ML tools for predicting trace metal concentrations, especially where monitoring data are extensive but mechanistic parameterization is uncertain. Comparative analyses indicate that neural networks can achieve coefficients of determination approaching 0.99 for selected metals, while RF models often demonstrate superior robustness and generalization across heterogeneous datasets [13,14]. These methods have proven particularly effective for identifying localized contamination hotspots, reconstructing missing observations, and elucidating complex interactions between hydrological and chemical predictors [15,16].

A growing body of evidence underscores the expanding application of ML frameworks for predicting dissolved trace elements and associated health risks in riverine systems [17] and drinking-water distribution networks [18]. Spatiotemporal ML analyses increasingly enable robust source attribution and risk assessment in major river basins, while explainable modeling approaches enhance interpretability in groundwater trace element evaluation [19]. These developments underscore the transition of ML from purely predictive tools toward integrative frameworks capable of supporting environmental decision-making.

Beyond conventional concentration forecasting, recent contributions demonstrate the capacity of ML frameworks to reconstruct unobserved system states from accessible measurements, including vertical coupling between hypolimnetic and epilimnetic layers in stratified lakes [20] and long-term analyses demonstrating how hydrodynamic–biogeochemical regime evolution shapes eutrophication trajectories [21]. These advances highlight the capacity of ML models not only to approximate concentration patterns but also to capture latent process interactions within complex aquatic systems.

Recognizing the complementary strengths of mechanistic and empirical approaches, recent studies increasingly employ hybrid modeling strategies. Techniques combining wavelet decomposition with neural networks, principal component analysis with multilayer perceptrons, or geographically weighted regression with ML have been shown to enhance long-term prediction accuracy and spatial resolution in systems influenced by mixed point and diffuse pollution sources [22,23,24]. Such hybrid frameworks are particularly valuable in urban and peri-urban streams, where industrial effluents, agricultural runoff, and domestic discharges interact over short spatial extents and generate pronounced spatial nonstationarity.

Across these modeling paradigms, a convergence emerges regarding the most influential predictors of trace metal distribution. Large-scale syntheses consistently identify hydrological regime, sediment dynamics, and water chemistry as the primary controls, with land use and point-source inputs acting as secondary determinants that shape local concentration gradients [25]. This predictor structure aligns with the metals most frequently modeled in stream ecosystems—zinc and lead, followed by copper, cadmium, iron, manganese, chromium, nickel, arsenic, and mercury (Hg)—each associated with characteristic anthropogenic and geogenic sources [26,27]. Hg represents a particular challenge because of its complex speciation and transformation pathways, which introduce additional uncertainty into both spatial mapping and temporal forecasting [28].

Spatial prediction studies further demonstrate that model performance is scale-dependent, ranging from meter-scale site assessments to reach- and watershed-scale representations. High-resolution mapping frequently reveals sharp concentration gradients downstream of industrial and urban outfalls, whereas broader-scale analyses capture diffuse loading patterns across entire catchments [29,30,31]. Among geostatistical tools, ordinary kriging has repeatedly been shown to outperform alternative interpolation methods in terms of prediction error, supporting its widespread adoption for spatial inference when monitoring density is limited [32,33].

Temporal prediction is equally critical because metal concentrations respond dynamically to seasonal hydrology, episodic storm events, and long-term environmental change. Modeling efforts span time scales from seconds to minutes for spill simulations, to hours and days for storm-driven resuspension, and to years or decades for trend analysis and climate-change scenarios [34,35,36]. Dynamic modeling approaches dominate because they are essential for reproducing dry-season concentration maxima, storm-related pulses, and climate-driven shifts in flux-weighted concentrations, whereas steady-state approaches are largely restricted to baseline characterization [27,37].

Despite substantial methodological advances, persistent limitations remain across computational studies. Data scarcity continues to constrain calibration and validation, particularly in urban streams characterized by sparse or irregular monitoring networks. Moreover, uncertainty quantification is often insufficiently developed, limiting confidence in projections beyond the calibration domain and under future climatic or land-use scenarios [25,38]. These challenges highlight the importance of well-designed empirical datasets that explicitly capture variability across monitoring locations and seasonal contrasts.

Within this broader modeling context, the Ova Stream (Ankara, Türkiye) represents a characteristic urban stream exposed to sustained industrial, agricultural, and residential pressures along its approximately 36 km course. Previous investigations in the basin have focused on biological quality and selected physicochemical parameters [39]; however, an integrated assessment of temporal variability in nitrogen and phosphorus fractions, together with multi-element trace metal profiles, has not yet been undertaken. Establishing a temporally resolved dataset, supported by observations across multiple monitoring stations, provides an essential empirical basis for evaluating and implementing predictive modeling frameworks in a complex, human-impacted lotic system.

## 2. Materials and Methods

### 2.1. Description of the Study Area

Ova Stream constitutes a principal northern tributary of the Ankara Stream within the Sakarya River Basin and is situated in the Mürted Plateau of Central Anatolia (northwest of Ankara). The stream extends approximately 36 km from its headwaters in the Kahramankazan district to its confluence with the Çubuk Stream in the Sincan district, forming the primary drainage axis of the Mürted Plateau. Hydrologically, the system integrates upstream inflows from the Pazar and Mera Streams, which discharge into the Kurtboğazı Dam and influence the headwater regime of the basin. In its lower reach, the Zir Stream within the Zir Valley enters the system. The Zir Valley, approximately 8 km in length and located about 30 km west of Ankara, is geomorphologically defined by a deeply incised valley morphology carved into the plateau surface. The watershed is intensively utilized and subject to sustained anthropogenic influence, including irrigation abstraction, aggregate extraction, industrial activities, livestock farming, and localized wastewater inputs. These pressures define the modified hydro-environmental context within which trace metal dynamics were evaluated. Within this setting, the Ova Stream sub-basin exhibits distinct hydrogeomorphological characteristics that further define system behavior. The Ova Stream sub-basin is situated within the Mürted Plateau (northwest of Ankara) and forms part of the Sakarya River Basin at an average elevation of approximately 890 m a.s.l. The stream drains a Quaternary alluvial corridor approximately 1–1.5 km in width, composed predominantly of sand and gravel deposits with an average thickness of 25–30 m, forming hydraulically productive aquifers. The groundwater potential of the sub-basin is estimated at approximately 21.5 hm^3^/year. At the regional scale, the Sakarya Basin contributes approximately 2900 hm^3^/year to total discharge, while total annual provincial outflow reaches ~5430 hm^3^/year. Geologically, the watershed is dominated by volcanic formations (andesite, basalt, tuff, agglomerate), Bilecik limestone units, and Pliocene marl–conglomerate sequences. Land use is characterized by intensive agriculture (46%), forested areas (17%), pasture/meadow lands (16%), and settlement/other uses (21%). These hydrogeomorphological characteristics define the structural framework governing surface–groundwater interaction and trace metal transport within the Ova Stream system. Accordingly, the sampling framework was designed to represent integrated system conditions under cumulative pressures.

**Figure 1:**
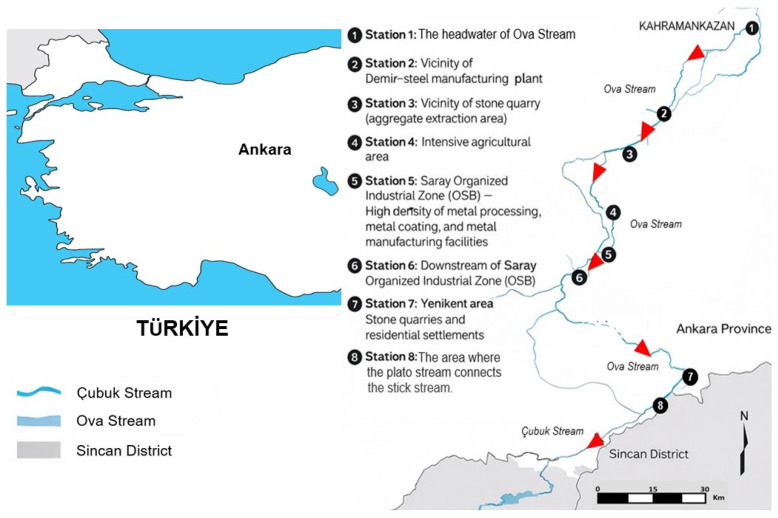
Overview of the study area and placement of sampling stations along the Ova Stream continuum. The red arrowhead illustrates the downstream direction of flow.

To ensure that sampled conditions were representative of the range of anthropogenic influences acting on the system, eight sampling stations were selected following detailed preliminary field surveys. While the primary analytical emphasis was placed on temporal variability, spatial heterogeneity was incorporated at the sampling-design level. Station identity was not included as an explicit spatial predictor, and no geostatistical interpolation was performed; instead, spatial variation is represented indirectly through site-specific hydrochemical and land-use characteristics. The geographic coordinates of all sampling stations were recorded using a Global Positioning System to provide accurate spatial documentation of the study area (Figure 1).

### 2.2. Sampling Strategy and Field Measurements

Field sampling was undertaken during two hydrologically contrasting periods corresponding to the dry (July) and wet (November) seasons, following established surface-water sampling protocols. At each monitoring station, duplicate surface-water grab samples were collected during each campaign to enhance analytical robustness. In situ measurements of water temperature, pH, electrical conductivity (EC), and dissolved oxygen (DO) were obtained immediately at each station using a portable multiparameter probe (YSI Inc., Yellow Springs, OH, USA) in combination with a pH meter. Upon completion of field measurements, water samples were maintained under cold-chain conditions (≈4 °C) and transported to the laboratory for subsequent physicochemical and chemical analyses.

### 2.3. Sample Preservation and Handling Procedures

Prior to chemical analyses, samples underwent gentle homogenization and were processed in accordance with standardized laboratory handling protocols to minimize potential contamination, matrix interferences, and analytical bias, thereby maintaining sample integrity and analytical reliability throughout the pre-analytical phase.

### 2.4. Analytical Procedures for Nitrogen and Phosphorus Fractionation

Concentrations of nitrogen and phosphorus fractions were quantified using standard spectrophotometric methods in accordance with procedures outlined by APHA [40]. Ammonia (NH_3_) was determined using the Nesslerization method, nitrite (NO_2_^−^) was measured via the α-naphthylamine–sulfanilic acid method, and nitrate (NO_3_^−^) was quantified using the brucine–sulfate method.

Total phosphorus (TP) was determined following persulfate digestion, during which organically bound and particulate phosphorus fractions were quantitatively converted to orthophosphate. The released orthophosphate was subsequently measured spectrophotometrically using the ascorbic acid method. Total orthophosphate (TOP) concentrations were likewise quantified using the ascorbic acid method. In addition, soluble reactive phosphorus (SRP)—operationally defined as the dissolved orthophosphate fraction—was determined following filtration of water samples through 0.45 µm membrane filters, in accordance with APHA [41]. All spectrophotometric measurements were performed using a UV–Vis spectrophotometer (UV-1800, Shimadzu Corp., Kyoto, Japan).

### 2.5. Instrumental Analysis of Trace Metals by Inductively Coupled Plasma–Mass Spectrometry (ICP-MS)

In accordance with TS EN ISO 15587-1 [42] and TS EN ISO 15587-2 [43] standards, water samples were filtered through 0.45 µm polyvinylidene fluoride membrane filters to remove particulate material, transferred into single-use polyethylene tubes to minimize the risk of contamination, and subsequently subjected to microwave-assisted acid digestion to achieve complete dissolution of the aqueous matrix and efficient extraction of trace metal constituents.

The concentrations of chromium (Cr), nickel (Ni), copper (Cu), arsenic (As), cadmium (Cd), mercury (Hg), and lead (Pb) were determined using an ICP-MS system (Agilent 7500cx, Agilent Technologies, Santa Clara, CA, USA) equipped with an inductively coupled plasma ion source and a quadrupole mass spectrometric analyzer, with analyses conducted in accordance with TS EN ISO 17294-1 [44] and TS EN ISO 17294-2 [45] guidelines. The instrument was operated at a radiofrequency power of 1550 W, with plasma, auxiliary, carrier, and make-up gas flow rates of 15, 1, 0.9, and 0.15 L min^−1^, respectively. Sample introduction was achieved using a concentric nebulizer coupled to a water-cooled (2 °C) double-pass spray chamber, employing nickel sampler and skimmer cones. The system was operated at a mass resolution of 0.8 u, with an integration time of 0.1 s per isotope.

Quantification was achieved by external calibration using multi-element standard solutions prepared at predefined concentration levels for each target analyte. Quality control standards and procedural blanks were included in each analytical batch to monitor instrumental performance and assess potential contamination.

### 2.6. Dataset Description

The dataset was derived from a station-based, season-resolved sampling design in which multiple physicochemical parameters, nutrient fractions, and dissolved trace metals were measured concurrently at each sampling event. While the analytical dataset used for machine-learning modeling comprises an expanded multivariate feature–response structure, the number of independent spatiotemporal sampling units remains limited to the underlying station–season sampling framework. The input variables consist of fundamental physicochemical characteristics, including pH, water temperature (°C), dissolved oxygen (DO, mg L^−1^), and electrical conductivity (EC, µS cm^−1^), which characterize the overall hydrochemical conditions of the aquatic environment. Inorganic nitrogen forms, including NH_3_, NO_2_^−^, and NO_3_^−^, were incorporated to characterize key biogeochemical transformations and anthropogenic influences governing nitrogen cycling dynamics.

Furthermore, phosphorus fractions, comprising TP, TOP, and SRP were incorporated to characterize variations in phosphorus partitioning and to explore their potential interactions with trace metal behavior under differing hydrological conditions. The dataset additionally included concentrations of trace metals and metalloids, Cr, Ni, Cu, As, Cd, Hg, and Pb, which were designated as target variables in the regression analysis.

A categorical period variable was incorporated to denote seasonal circumstances, facilitating the analysis of temporal variability across sampling intervals (e.g., wet season and dry season). All variables were measured simultaneously for each observation, ensuring uniformity among predictors and response variables.

Before model creation, the dataset was scrutinized for absent or infinite values, and none were identified. The entire dataset was later utilized for ML investigations, encompassing regression modeling, performance evaluation, and feature importance assessment.

### 2.7. Machine Learning (ML) Models

Multiple regression-based ML algorithms were employed and thoroughly compared to model the intricate and nonlinear interactions between physicochemical parameters and trace metal concentrations. The chosen models encompass a variety of learning procedures, including ensemble methods, kernel regression, and parametric techniques, facilitating a comprehensive assessment of predicted performance across various metals.

#### 2.7.1. Random Forest (RF) Regression

RF regression is an ensemble learning technique that generates numerous decision trees by bootstrap sampling and random feature selection, subsequently consolidating their outputs through averaging [46]. This technique efficiently decreases variance and alleviates overfitting while encapsulating intricate nonlinear relationships among predictors. RF regression was utilized as the principal benchmark model for predicting trace metals due to its resilience and interpretability.

#### 2.7.2. Extreme Gradient Boosting (XGBoost) Ensemble Regression

XGBoost is a sophisticated ensemble technique based on boosting that constructs decision trees in a sequential manner, with each tree refined to address the residual mistakes of preceding rounds [47]. XGBoost incorporates regularization, shrinkage, and efficient gradient-based optimization, rendering it especially useful for high-dimensional and nonlinear regression challenges. This study employed XGBoost regression to assess its ability to model nuanced fluctuations in trace metal concentrations.

#### 2.7.3. Radial Basis Function (RBF) Regressor

RBF regression is a kernel-based learning method that characterizes nonlinear relationships by transforming input variables into a higher-dimensional feature space through distance-based radial functions. Initially shown in adaptive network frameworks, RBF-based models are adept at modeling intricate nonlinear processes characterized by smooth functional behavior [48,49]. In environmental modeling, RBF regression has been extensively utilized to elucidate nonlinear relationships between physicochemical factors and pollutant concentrations. This study incorporated RBF regression to assess the efficacy of kernel-based methods in comparison to ensemble tree techniques.

#### 2.7.4. Polynomial Regression (PR)

PR served as a parametric baseline to evaluate if trace metal concentrations can be sufficiently represented by low-order nonlinear trends in the predictor space. This method involves augmenting the original predictors with polynomial terms (such as squares and interaction terms), and the resultant model is fitted using ordinary least squares within the generic linear modeling framework. While PR may accommodate specific forms of curvature, it is constrained in its ability to depict intricate, non-additive interactions as compared to adaptable nonparametric models like tree ensembles. Consequently, PR was incorporated to establish an interpretable reference model and to evaluate the degree to which nonlinearities in the data may be approximated by polynomial feature expansions [50,51]

### 2.8. Model Evaluation Metrics

The dataset was partitioned into training and testing subsets at the level of modeling records derived from the multivariate feature–response matrix using a stratified random sampling strategy. Stratification ensured proportional representation of station–season combinations in both subsets, while random selection was performed within each stratum.

All regression models were trained exclusively on the training subset, and predictive performance was evaluated on the corresponding test subset. This hold-out validation approach enables a consistent comparison of model performance within the observed spatiotemporal envelope and prevents information leakage between training and evaluation phases. Model performance was subsequently assessed using complementary evaluation metrics, as detailed below. The predictive performance of the regression models was evaluated using multiple complementary metrics to quantify goodness-of-fit, prediction accuracy, and relative error magnitude. Let $y_{i}$ denote the observed value, ${\hat{y}}_{i}$ the predicted value, $\bar{y}$ the mean of observed values, and n the total number of observations.

The coefficient of determination $\left(R^{2}\right)$ represents the proportion of variance in the observed data explained by the model and is defined as

$$R^{2}=1-\frac{\sum_{i=1}^{n}{\left(y_{i}-{\hat{y}}_{i}\right)}^{2}}{\sum_{i=1}^{n}{\left(y_{i}-\bar{y}\right)}^{2}}$$

Higher $R^{2}$ values indicate stronger agreement between predicted and observed concentrations.

The mean absolute error measures the average magnitude of prediction errors without considering their direction:

$$\mathrm{MAE}=\frac{1}{n}\overset{n}{\underset{i=1}{\sum }}|y_{i}-{\hat{y}}_{i}|$$

MAE provides an intuitive measure of model accuracy expressed in the same units as the target variable.

The root mean squared error penalizes larger deviations more strongly by squaring residuals prior to averaging:

$$\mathrm{RMSE}=\sqrt{\frac{1}{n}\overset{n}{\underset{i=1}{\sum }}{\left(y_{i}-{\hat{y}}_{i}\right)}^{2}}$$

RMSE is particularly sensitive to extreme prediction errors and reflects model robustness.

The mean absolute percentage error expresses prediction accuracy in relative terms:

$$\mathrm{MAPE}=\frac{100}{n}\overset{n}{\underset{i=1}{\sum }}|\frac{y_{i}-{\hat{y}}_{i}}{y_{i}}|$$

MAPE enables performance comparison across trace metals with different concentration ranges.

## 3. Results

### 3.1. Descriptive Statistics of Physicochemical Parameters

Descriptive statistical summaries of physicochemical parameters, nitrogen and phosphorus forms, and trace metal concentrations characterizing the wet-season hydrological period are reported in Table 1. The wet season dataset is characterized by relatively stable pH conditions, moderate EC, and a wide range of DO concentrations, reflecting heterogeneous hydrological and biogeochemical conditions. Nitrogen (NH_3_, NO_2_^−^, and NO_3_^−^) and phosphorus (TP, TOP, SRP) fractions exhibited notable variability, indicating fluctuating dynamics during the sampling period. Trace metals showed distinct concentration ranges and dispersion characteristics, with differences in variability reflected by standard deviation, skewness, and kurtosis values. No missing or infinite values were detected in the wet season dataset, confirming data completeness and suitability for subsequent modeling analyses.

To account for seasonal variability in physicochemical conditions and trace metal concentrations, descriptive statistics were additionally evaluated for the dry season sampling period (Table 2). Seasonal differentiation of the dataset reduces masking effects associated with aggregated summaries and provides a clearer representation of the environmental context underlying subsequent modeling analyses.

A comparative evaluation of the descriptive statistics for the wet-season (Table 1) and dry-season (Table 2) datasets reveals pronounced season-dependent differentiation in both physicochemical conditions and trace metal distributions. Several variables displayed expanded concentration envelopes and enhanced statistical dispersion during the dry season, as evidenced by elevated standard deviation values, indicating increased system variability under reduced flow conditions. Among the trace metals, Cr and Cd exhibited the most conspicuous seasonal divergence, characterized by higher mean concentrations coupled with greater dispersion during the dry period. In contrast, Ni demonstrated comparatively muted seasonal variability, suggesting a more temporally stable geochemical behavior across hydrological conditions.

### 3.2. Seasonally Informed Regression Performance of Physicochemical Parameters

The regression performance of ML models was assessed for essential physicochemical and nitrogen fractions. Various regression algorithms were evaluated for each parameter, and model efficacy was measured using *R*^2^ in conjunction with error-based metrics.

To ensure clarity and eliminate redundancy, only the optimal regression model for each parameter is presented, determined by the comprehensive assessment of *R*^2^, RMSE, and MAE. Table 3 presents a summary of the selected models together with their respective performance indicators.

The findings demonstrate that ensemble-based and nonlinear regression methods consistently attained good prediction accuracy across the majority of factors. Specifically, physicochemical factors like pH, temperature, and electrical conductivity were modeled with minimal prediction errors, but nitrogen-related parameters displayed slightly greater variability but still demonstrated strong predictive performance.

The initial investigation of seasonal variability in physicochemical and nitrogen-related parameters employed descriptive and distribution-based methods to discern overall trends before proceeding to regression modeling. Box plot representations were utilized to illustrate variations between wet and dry seasons, facilitating the evaluation of central tendency, dispersion, and potential outliers among parameters with diverse measurement scales.

**Figure 2:**
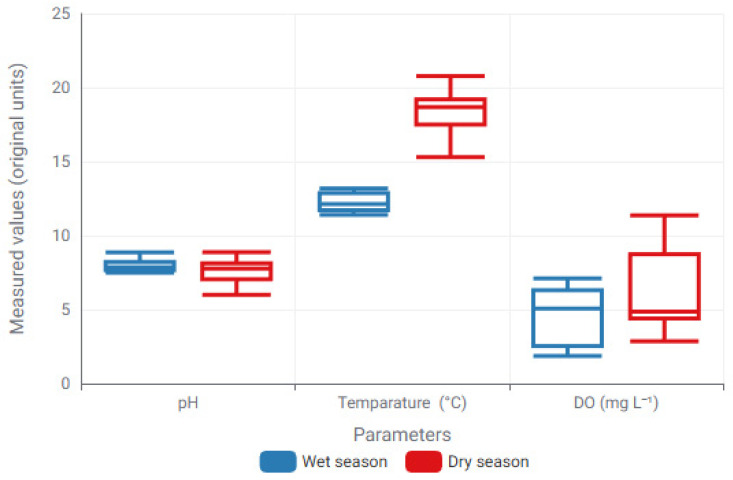

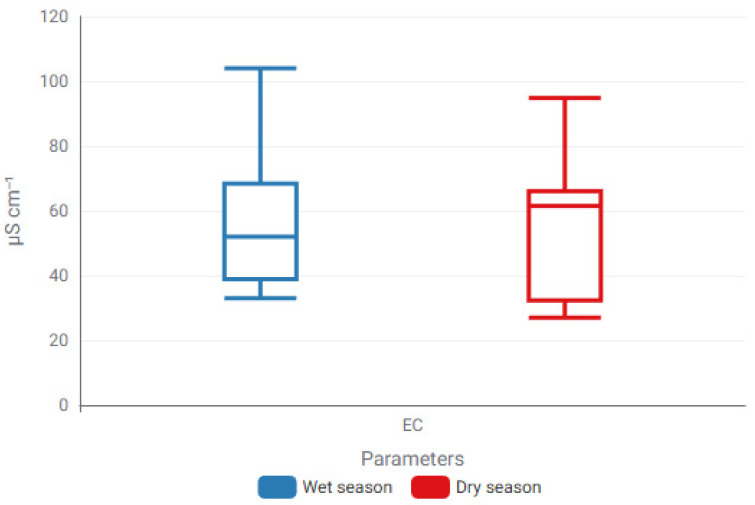
Seasonal modulation of physicochemical water-quality parameters illustrated through box-plot distributions for wet and dry periods. Boxes denote the interquartile range (IQR), central lines indicate median values, and whiskers represent the minimum and maximum observations excluding outliers. Box-plots display raw measurements in their original units; absolute magnitudes are not intended for direct cross-parameter comparison.

**Figure 3:**
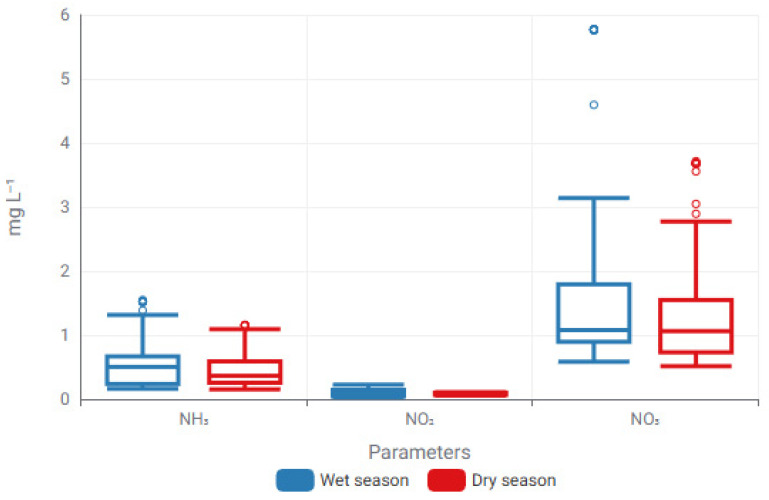
Seasonal modulation of nitrogen fractions illustrated through box-plot distributions for wet and dry periods. Boxes denote the interquartile range (IQR), central lines indicate median values, and whiskers represent the minimum and maximum observations excluding outliers (Circles represent mild outliers).

Figure 2 and Figure 3 illustrate the presence of unique seasonal patterns among the monitored variables. Temperature and DO had distinct seasonal variation, reflecting climatic and hydrological factors, whereas EC showed greater change throughout seasons. Nitrogenous forms often exhibited lower concentration ranges, with NO_3_^−^ demonstrating heightened dispersion in the wet season, indicating seasonally dependent nitrogen dynamics.

Building upon these seasonally driven patterns, ML–based regression models were subsequently applied to quantify the predictive relationships between environmental parameters. The regression performance of the best-performing models for each parameter is summarized in Table 3.

These exploratory findings offer crucial context for the ensuing ML-based regression analyses by emphasizing parameter-specific seasonal behavior and variability, thereby facilitating informed interpretation of model performance and predictive sensitivity.

Building upon the seasonally informed analytical framework, the regression performance of ML models was further evaluated for phosphorus fractions, specifically TP, TOP, and SRP, which collectively capture variability in phosphorus partitioning and transformation processes. Given the pronounced temporal and environmental variability governing phosphorus dynamics, multiple regression approaches were employed to resolve both linear and nonlinear response structures. Consistent with the physicochemical modeling framework, model performance was assessed using *R*^2^ in conjunction with complementary error-based metrics. To enhance interpretability and avoid redundancy, only the best-performing regression model for each phosphorus fraction is reported, selected through comparative evaluation of predictive accuracy and error magnitude. The relevant findings are encapsulated in Table 4.

The regression models exhibited robust prediction capacity for phosphorus fractions, while performance differed among parameters, indicating variations in their inherent variability and responsiveness to environmental factors. These results demonstrate that ML-based regression methods are proficient in modeling parameters associated with phosphorus fractions and offer a dependable foundation for further analysis.

Table 4 summarizes that the regression performance of ML models for phosphorus fractions demonstrated variability dependent on parameters. Among the assessed indicators, TOP exhibited the greatest predictability, with the XGBoost Tree Ensemble model attaining a substantial coefficient of determination and minimal prediction errors. This signifies a robust and consistent correlation between TOP and the input variables included in the regression model.

TP and SRP displayed moderate predictive performance, consistent with the nonlinear, context-dependent behavior of phosphorus partitioning in the stream system. For TP, RF and SRP ensemble modeling provided the most balanced performance across evaluation metrics. The divergence in model performance across phosphorus fractions highlights the need for parameter-specific modeling strategies, rather than reliance on a uniform regression framework.

**Figure 4:**
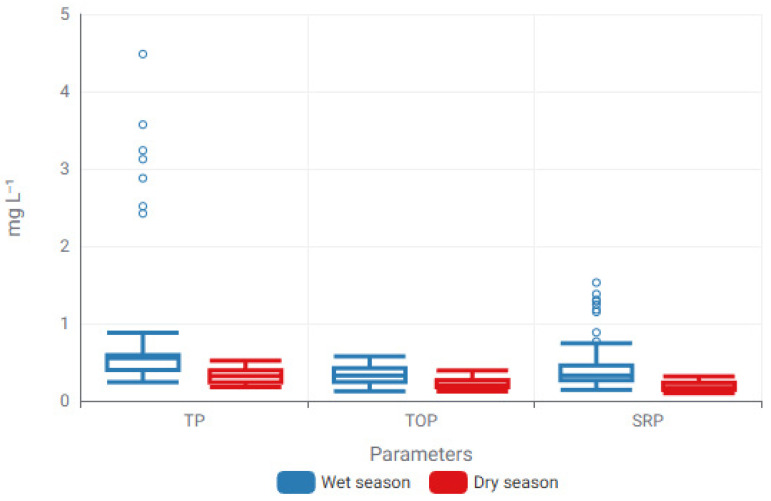
Seasonal modulation of phosphorus fractions illustrated through box-plot distributions for wet and dry periods. Boxes denote the interquartile range (IQR), central lines indicate median values, and whiskers represent the minimum and maximum observations excluding outliers (Circles represent mild outliers).

Seasonal organization of phosphorus fractions was further examined using box-plot analysis to resolve temporal variability. Distributional patterns of TP, TOP, and SRP under wet- and dry-season conditions are presented in Figure 4, offering complementary insight into the seasonal modulation underlying observed differences in model behavior.

Box-plot analysis revealed pronounced seasonal modulation of phosphorus fractions across the study period. TP exhibited substantial variability during the wet season, characterized by a broadened interquartile range and the presence of high-concentration extremes, whereas dry-season values displayed comparatively constrained dispersion. TOP showed a consistent seasonal shift, with elevated median concentrations under wet-season conditions, reflecting enhanced phosphorus mobilization during periods of increased hydrological connectivity. Similarly, SRP demonstrated increased dispersion and higher concentration levels during the wet season, indicative of seasonally driven changes in phosphorus availability and transformation processes.

**Figure 5:**
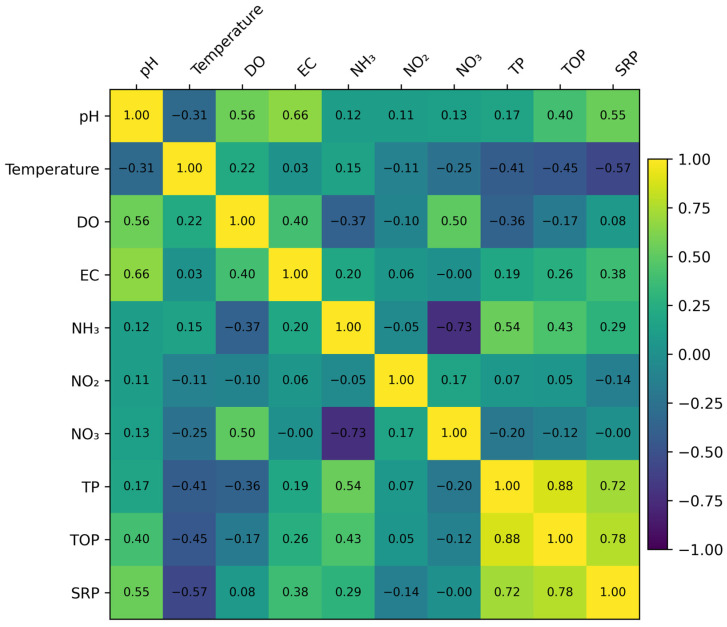
Spearman rank correlation heatmap depicting multivariate associations among physicochemical parameters, inorganic nitrogen forms, and phosphorus fractions. Color intensity denotes the magnitude and direction of correlation coefficients, elucidating coupled physicochemical relationships that structure the observed seasonal variability.

To further interrogate the environmental controls underlying these seasonal patterns, correlation analysis was employed to resolve interdependencies among measured variables. While box-plot analysis provides a robust depiction of distributional spread and seasonal amplitude, it does not explicitly capture multivariate relationships within the physicochemical system. Accordingly, Spearman rank correlation analysis was applied to examine associations among physicochemical parameters, nitrogen and phosphorus fractions (Figure 5). This multivariate perspective enables a more integrated interpretation of how coupled physicochemical interactions structure seasonal phosphorus behavior, thereby furnishing a process-informed context for the subsequent ML-based modeling analyses.

### 3.3. Performance of ML Models for Trace Metal Prediction Under Seasonal Conditions

RF regression, RBF regression, PR, and XGBoost tree ensemble regression approaches were evaluated to predict trace metal concentrations using physicochemical and environmental predictors. Model performance was quantified using *R*^2^ together with error-based metrics, including MAE, RMSE, and MAPE. For each target metal, the optimal algorithm was selected based on a joint assessment of explanatory power and prediction error. Ensemble-based and nonlinear models achieved high predictive performance across metals, with *R*^2^ values generally exceeding 0.95 and low associated error metrics, indicating strong agreement between observed and predicted concentrations (Figures S1–S7, Supplementary Material).

Model evaluation for Cr revealed consistently high agreement between observed and predicted concentrations across all tested algorithms (Table 5). The RF model provided the most accurate predictions, yielding the lowest absolute and squared error metrics among the competing approaches. Error sign analysis indicated minimal directional bias, with mean signed differences remaining close to zero, confirming balanced model performance.

For Ni, all evaluated regression models demonstrated high predictive performance, with *R*^2^ values exceeding 0.96 (Table 6). Among the tested algorithms, RF regression achieved the highest explanatory power and the lowest prediction errors, as reflected by minimal MAE, RMSE, and MAPE values. The small magnitude of mean signed differences across models indicates negligible systematic bias in Ni predictions.

Prediction results for Pb indicate a clear differentiation in model performance across the evaluated algorithms (Table 7). The RF regressor achieved the highest goodness-of-fit and the lowest absolute and squared error metrics, outperforming both linear and discretization-based approaches. Although alternative models captured a substantial portion of variance, their higher error values suggest reduced precision in Pb estimation. Mean signed differences were consistently close to zero, indicating negligible systematic bias across all models.

For Cd, model performance exhibited pronounced variability depending on the applied regression technique (Table 8). Ensemble-based and kernel-based approaches yielded superior explanatory power compared to polynomial models. The RF algorithm provided the most favorable balance between variance explanation and prediction error, whereas the RBF regressor achieved comparable goodness-of-fit with slightly higher error metrics. Across all models, mean signed differences remained minimal, suggesting stable prediction behavior without directional bias.

As concentration predictions demonstrated consistently high agreement between observed and modeled values across all regression approaches (Table 9). RF and XGBoost models achieved the highest predictive accuracy, as reflected by lower MAE and RMSE values relative to polynomial and kernel-based regressors. Despite differences in error magnitude, all models maintained low mean signed differences, indicating robust and well-balanced predictive performance for As.

The predictions of Cu content exhibited a consistently robust correlation between observed and modeled values across all assessed regression methodologies (Table 10). Of the evaluated models, the XGBoost Tree Ensemble attained the greatest predictive accuracy, evidenced by the highest *R*^2^ and the lowest error metrics. RF regression demonstrated strong performance, while polynomial and kernel-based regressors revealed somewhat greater prediction errors.

Notwithstanding variations in error magnitude between models, the mean signed difference values remained modest, signifying negligible systematic bias in Cu forecasts. The findings affirm that ML–based regression models can consistently quantify Cu concentration fluctuation, with ensemble methods demonstrating enhanced predictive efficacy.

Hg concentration predictions demonstrated strong agreement between observed and modeled values across most regression approaches (Table 11). Among the evaluated models, RF regression achieved the highest predictive accuracy, as reflected by the highest *R*^2^ value and minimal prediction errors. RBF and PR models also yielded robust performance, whereas the XGBoost Tree Ensemble showed comparatively reduced explanatory power for Hg prediction. These results indicate that ensemble-based models are particularly effective in modeling Hg concentrations despite the low-magnitude and noise-sensitive nature of trace metal data.

The regression results demonstrate that predictive performance varies across trace metals, with ensemble-based approaches generally yielding superior accuracy and lower error metrics. While RF regression consistently achieved the best balance between goodness-of-fit and prediction error for most metals, alternative algorithms provided competitive performance depending on the target variable. These findings highlight the metal-specific nature of model behavior and motivate further examination of the relative contribution of input variables, which is addressed through feature importance analysis in the following section.

**Figure 6:**
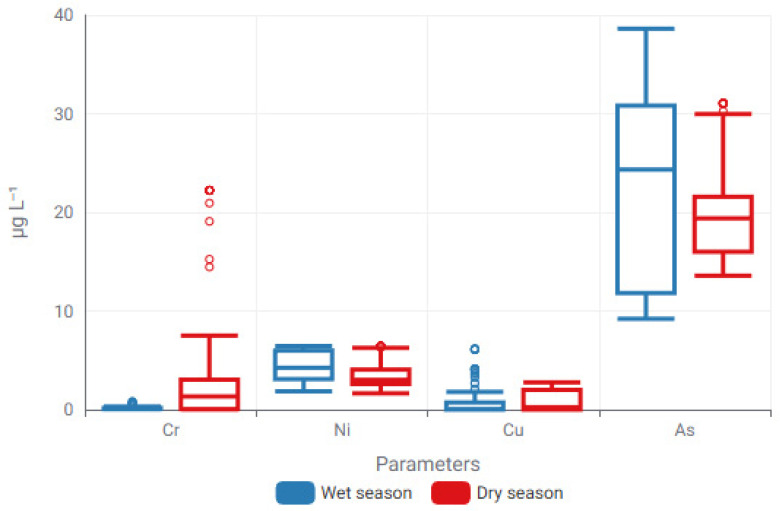
Seasonal modulation of trace metal concentrations comprising Cr, Ni, Cu and As illustrated through box-plot distributions for wet and dry periods. Boxes denote the interquartile range (IQR), central lines indicate median values, and whiskers represent the minimum and maximum observations excluding outliers (Circles represent mild outliers).

**Figure 7:**
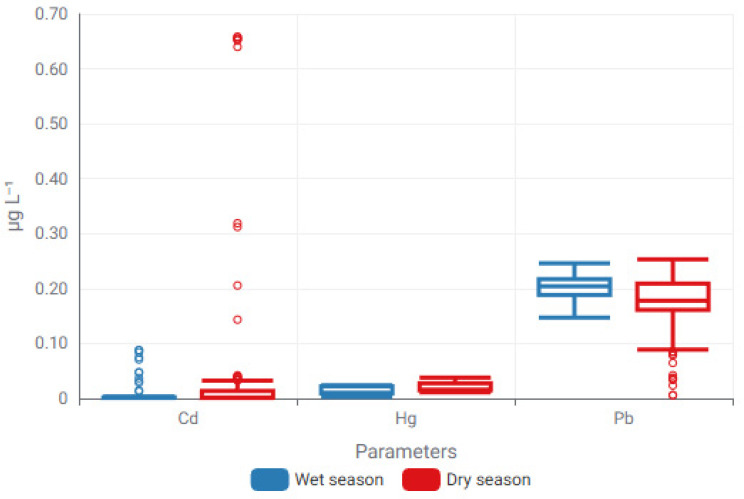
Seasonal modulation of trace metal concentrations comprising Cd, Hg and Pb illustrated through box-plot distributions for wet and dry periods. Boxes denote the interquartile range (IQR), central lines indicate median values, and whiskers represent the minimum and maximum observations excluding outliers (Circles represent mild outliers).

Seasonal modulation of trace metal concentrations was further examined through box-plot analysis, enabling direct comparison of distributional characteristics between wet and dry hydrological periods. The resulting concentration envelopes and dispersion patterns, presented in Figure 6 and Figure 7, provide complementary evidence for hydrologically driven variability in trace metal behavior.

The graph demonstrates significant seasonal variations in both central tendency and dispersion among metals, emphasizing element-specific reactions to seasonal hydrological and environmental processes.

**Figure 8:**
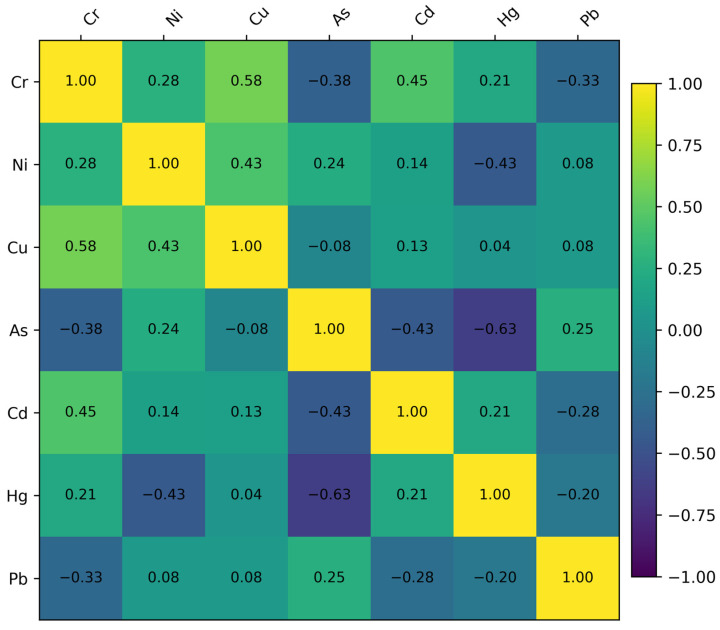
Spearman rank correlation heatmap depicting pairwise relationships among trace metal concentrations (Cr, Ni, Cu, As, Cd, Hg, and Pb). Color intensity represents the strength and direction of correlation, revealing metal-specific association patterns that reflect shared geochemical controls, source characteristics, and mobility behavior underlying the seasonal variability observed in trace metal distributions.

The box plot analysis indicates that seasonal variations in trace metal distributions are influenced by factors beyond just seasonal forces. While box plots clearly depict dispersion, median variations, and outlier behavior, they do not explicitly elucidate the relationships among individual elements. A Spearman rank correlation heatmap was utilized to analyze element–element correlations, facilitating the identification of shared geochemical behavior, likely common sources, and differing mobility patterns among trace metals (Figure 8).

### 3.4. Feature Importance Analysis for Trace Metal Prediction

To enhance the interpretability of the ML framework, a feature importance analysis was conducted for all investigated trace metals using tree-based regression models. Variable importance was quantified using gain-based metrics, which measure the relative contribution of each predictor to reducing prediction error during model training. To ensure consistency and comparability across metals, feature importance rankings were evaluated separately for each target metal and subsequently synthesized to identify dominant physicochemical and environmental drivers.

Across all trace metals, the analysis indicates that predictive performance is influenced by a combination of physicochemical conditions and co-occurring trace metal concentrations. Predictors associated with pH, temperature, and redox-sensitive parameters consistently ranked among the most influential variables, whereas nitrogen-related parameters exhibited moderate contributions. On the other hand, EC and phosphorus fractions generally showed lower relative importance. These results highlight the multivariate nature of trace metal prediction within the studied stream system. As a representative example, the gain-based feature importance ranking for Cr is presented in Table 12, illustrating the relative influence of environmental predictors on model performance.

Following the chromium case, feature importance analysis was further examined for Ni to assess whether similar environmental controls govern its prediction. The gain-based importance ranking for Ni is summarized in Table 13.

As presented in Table 14, feature importance analysis for Pb indicates that prediction accuracy is primarily governed by interactions with other trace metals and redox-related parameters.

Feature importance analysis was further applied to Cd to identify the dominant predictors governing its variability. The gain-based importance ranking for Cd prediction is summarized in Table 15.

The detailed gain-, weight-, and cover-based feature importance metrics for the metalloid As are reported in Table 16, providing a comprehensive quantitative assessment of predictor contributions.

Feature-importance analysis indicates that trace metal behavior in the investigated stream is structured by a heterogeneous constellation of environmental drivers, rather than by any single governing control. Variables associated with thermal conditions, dissolved oxygen, and nitrogen and phosphorus forms consistently emerge as dominant contributors, while the influence of co-occurring trace elements varies according to the metal under consideration. The gain-based importance hierarchies differ markedly among metals, demonstrating that each element is regulated by a distinct configuration of physicochemical and geochemical controls. These results establish that predictor–response relationships are inherently metal-specific and must be interpreted within a multivariate, process-resolved framework, rather than inferred from generalized or transferable dominance patterns.

## 4. Discussion

The Ova Stream dataset indicates a pronounced seasonal restructuring of background hydrochemistry that plausibly governs the mobility, partitioning, and apparent predictability of trace metals in this human-impacted lotic system, a pattern increasingly recognized as a critical challenge for freshwater sustainability under intensifying anthropogenic pressures and climate-related hydrological variability [1,2]. During the wet season, pH remains circumneutral to alkaline (7.49–8.87), EC is moderate (33–104 µS cm^−1^), and DO spans a wide range (1.86–7.12 mg L^−1^), reflecting heterogeneous hydrological mixing and alternating oxic–suboxic microenvironments that can modulate adsorption–desorption equilibria, redox-mediated transformations, and short-scale water–sediment exchange [6,7,9]. In the dry season, the hydrochemical envelope expands substantially (pH down to 5.90; DO up to 11.37 mg L^−1^), consistent with reduced dilution, stronger local controls, and intensified diel or reach-scale biogeochemical regulation under more stable hydraulics—conditions under which point-source influence and in-channel processing can become more apparent in measured concentrations [10,34,35,52]. Interpreting model outputs without explicitly accounting for this season-aware hydrochemical context risks misattributing predictability solely to algorithm choice alone rather than to a physically meaningful seasonal reorganization of the system, with direct implications for sustainability-oriented freshwater assessment frameworks [2].

Within that seasonal framework, the empirical results emphasize that metal behavior is strongly element-specific and consistent with changing dominance of dilution, source proximity, and sediment coupling. Cr and Cd show the strongest seasonal contrast, with markedly higher maxima and dispersion during the dry season (Cr max 22.23 µg L^−1^; Cd max 0.66 µg L^−1^), while Ni remains comparatively stable across seasons. This configuration is consistent with dry-season amplification of concentration signals through reduced dilution and increased sensitivity to localized inputs, a common feature of urban and peri-urban streams where industrial discharges and mixed effluents can dominate short reaches and produce sharp gradients [4,5,22], particularly in regions where a high proportion of surface waters are already classified as polluted [2]. In parallel, low-flow conditions can elevate the relative importance of sediment–water exchange, near-bed processes, and episodic resuspension events driven by local disturbances, producing sporadic concentration spikes for redox-sensitive and particle-associated elements [7,26,34]. By contrast, As exhibits relatively high concentrations in both seasons (wet mean 24.91 µg L^−1^; dry mean 19.71 µg L^−1^), implying either persistent source contributions (continuous discharges and/or stable geogenic background) or consistent control by sorption equilibria and buffering across hydrological periods. Similar persistence has been noted in catchments where apparent concentrations integrate ongoing inputs, sediment binding, and seasonally varying but compensating transport/retention processes [25,27,36]. Hg remains low in absolute magnitude yet shows seasonal shifts (wet mean 0.013 µg L^−1^; dry mean 0.020 µg L^−1^), consistent with the well-documented complexity of Hg speciation, transformation, and strong dependence on organic matter and particle dynamics—processes that frequently introduce additional uncertainty in predictive frameworks [28,53]. Together, the observed contrasts support a coherent interpretation in which dry-season conditions accentuate local gradients and episodic peaks via reduced dilution and stronger point-source control, whereas wet-season conditions enhance catchment connectivity and phosphorus forms variability, redistributing metals via runoff-driven transport and changing partitioning behavior [35,36,37].

Beyond seasonal restructuring, station-level variability further clarifies how localized anthropogenic activities shape longitudinal metal distributions along the stream continuum. The spatial distribution of trace metals across the eight sampling stations exhibits pronounced site-specific differentiation that closely corresponds to surrounding land-use configuration and source proximity. Stations 5 and 6, situated within the Saray Organized Industrial Zone, consistently displayed elevated concentrations of Cr, Cu, and As during the wet season, with Station 6 demonstrating a marked amplification of Cr under dry-season conditions. The magnitude and spatial confinement of these enrichments indicate localized emission dominance rather than progressive downstream accumulation. The pronounced dry-season intensification of Cr at Station 6 further underscores the role of hydrological contraction, whereby reduced dilution capacity enhances the expression of proximal industrial inputs.

By contrast, stations primarily influenced by agricultural and livestock activities (Stations 4, 7, and 8) exhibited enrichment patterns characterized by broader spatial distribution and comparatively moderate concentration magnitudes. Elevated Ni at Station 4 and the downstream increase in Cd at Station 8 are consistent with diffuse contributions mediated through runoff-driven transport, soil mobilization, and agricultural amendments. The signal observed at Station 8 appears to reflect cumulative integration of upstream industrial signatures with locally derived agricultural inputs, rather than the dominance of a single point source.

Importantly, the absence of a systematic longitudinal gradient across all metals indicates that the stream is not governed by uniform cumulative loading. Instead, metal distributions reflect superimposed anthropogenic pressures operating at multiple spatial scales. Industrial zones generate spatially discrete concentration anomalies, whereas agricultural landscapes contribute distributed enrichment modulated by hydrological connectivity and seasonal transport dynamics.

This station-level heterogeneity aligns with the spatial configuration of the study design. The eight stations were distributed along the ~36 km Ova Stream continuum to encompass contrasting anthropogenic settings, including industrialized zones, aggregate extraction areas, livestock operations, irrigated agricultural land, and residential settlements. Although geographic position was not incorporated as an explicit predictor and no formal geostatistical interpolation was undertaken, the sampling framework intentionally introduced structured environmental contrasts along the longitudinal axis of the stream. Within this design, spatial heterogeneity is not resolved as an independent geographic effect but is indirectly expressed through systematic variation in physicochemical parameters, nitrogen and phosphorus fractions, and co-occurring metals that differ among land-use contexts. Consequently, the models do not parameterize space per se; rather, they capture environmentally structured covariance patterns associated with location-specific anthropogenic settings. In this sense, longitudinal organization emerges as an embedded environmental signature rather than as an explicitly modeled spatial process. This interpretation is consistent with stream-network modeling evidence showing that predictive skill depends not only on algorithm choice but also on how effectively sampling design and covariates encode flow-connected structure and spatial heterogeneity [30,31,32,33,34,35,36,37,38,39,40,41,42,43,44,45,46,47,48,49,50,51,52,53,54]. Where geostatistical mapping is applied, kriging has frequently been shown to outperform alternative interpolation approaches under sparse monitoring conditions, although its performance remains strongly dependent on station density and variogram adequacy [31,32,33]. In the present context, the sampling framework is therefore best understood as introducing structured environmental contrasts along the stream continuum and as providing a basis for training predictive models within the observed monitoring envelope, rather than as a substitute for fully distributed geostatistical mapping. Hydrodynamic conditions further modulate the expression of this longitudinal variability by regulating dilution capacity, residence time, and mixing dynamics along the stream continuum. Under low-flow conditions, reduced dilution enhances reach-scale concentration anomalies, whereas higher flows promote redistribution through advection and resuspension processes. Reservoir-scale evidence similarly indicates that hydrodynamic configuration can substantially influence apparent metal gradients and associated risk signals [55]. Viewed in this integrated spatial–hydrodynamic context, the exceptionally strong ML performance observed across multiple targets is interpretable as the outcome of both robust algorithms and informative environmental structure. RF frequently provided the best balance of fit and error across metals, while XGBoost excelled for several physicochemical variables and for Cu (and in some cases As), consistent with broader findings that ensemble tree methods capture nonlinearities, threshold behavior, and interaction effects typical of contaminant systems governed by coupled hydrology and chemistry [13,14,56]. Numerous comparative studies likewise report that RF can outperform support vector machine and artificial neural network in heterogeneous environmental datasets, particularly when predictors are multicollinear or when response behavior is nonstationary across regimes [13,33,57]. At the same time, the high *R*^2^ values warrant careful interpretation. They likely reflect genuine structure but may also be inflated by seasonal clustering and shared covariance among predictors (e.g., temperature–DO–phosphorus fractions) and by inclusion of co-occurring metals that encode shared sources or co-transport pathways. This is not inherently problematic—co-metals can function as effective proxies for unmeasured process variables—but it shifts the meaning of “prediction” toward learning multivariate signatures rather than isolating mechanistic causality [16,20]. This distinction is particularly important given longstanding concerns that mechanistic models may misrepresent dynamics or overestimate concentrations when attenuation, hyporheic exchange, and sediment–water interactions are simplified, including the known limitations of equilibrium partitioning (Kd) approaches under dynamic conditions [7,9,12]. The present framework addresses these challenges differently: it leverages empirical structure rather than prescribing transport and fate, which is advantageous for forecasting under similar conditions, but it also increases the importance of transparent validation and uncertainty communication when extrapolation is contemplated [25,38]. Despite the coherent spatial–seasonal structure identified, several process-relevant variables—including suspended solids/turbidity, dissolved organic carbon (DOC), stream discharge, and sediment characteristics—were not directly measured. These factors exert fundamental control over metal partitioning, transport, and attenuation. Their absence implies that certain mechanistic inferences remain provisional. In particular, the predictive contribution of co-occurring metals may partly reflect shared source signatures or indirect encoding of unmeasured particulate or organic-mediated processes. Accordingly, the results are best interpreted as empirically robust within the sampled environmental envelope, while mechanistic attribution should remain cautious pending integration of direct hydrological and sedimentary covariates. Even within these limitations, the internal structure of predictor contributions remains informative.

The feature-importance results provide an additional process-oriented lens that strengthens interpretability and reinforces the element-specific nature of controls. Across targets, the prominence of temperature and dissolved oxygen supports their role as integrative proxies for seasonal hydrology, redox regime, and biogeochemical processing, which govern partitioning and mobility for multiple metals [6,7,35]. Nitrogen and phosphorus fractions emerge as important predictors for some metals, aligning with the role of runoff connectivity and phosphorus-associated processes that tend to intensify during wetter hydrological periods. Hybrid modeling strategies that combine signal decomposition or dimensionality reduction with ML have been proposed specifically to manage such seasonally varying, multicollinear regimes and to improve long-term prediction under nonstationarity [13,23,24]. The repeated emergence of co-occurring metals as influential predictors (e.g., Hg contributing strongly to As prediction; Hg and pH relevant for Cr) reflects the presence of shared input signatures and synchronized transport dynamics embedded within the dataset. Comparable “metal–metal” predictive utility has been reported in data-driven studies where anthropogenic sources and hydrological forcing generate coherent multivariate fingerprints [15], and such behavior also highlights a practical advantage: co-metals can compensate for missing process covariates (e.g., suspended solids, dissolved organic carbon). Nevertheless, it also implies that transferring models to different catchments or future periods with altered source mixtures may reduce performance unless the model is recalibrated or constrained by additional process information.

Within the wider computational modeling landscape, these findings support a complementary rather than competitive framing of mechanistic and data-driven approaches. Physically based models remain indispensable for scenario testing, regulatory load allocation, and assessing responses to management interventions, particularly when extrapolating beyond observed conditions [10,11,58]. Their accuracy improves when water chemistry variables governing speciation (pH, DO, EC and temperature) are integrated into transport formulations, but they remain sensitive to parameterization and representation of in-channel processes [7,9]. ML approaches, by contrast, excel when extensive monitoring data exist and when complex nonlinear interactions dominate, offering rapid prediction and hotspot screening in heterogeneous systems [14,16,59]. From an applied perspective, this supports two practical pathways: (i) the use of ensemble regressors for screening-level assessment, anomaly detection, and prioritization of targeted follow-up analyses; and (ii) the use of the assembled seasonally resolved dataset to calibrate or benchmark mechanistic frameworks applied in scenario analyses for comparable urban stream systems [11,53].

Methodologically, the high predictive performance should be interpreted as robust within the sampled spatio-temporal envelope, as model evaluation relied on a 70/30 train–test split designed to ensure balanced representation of sampling locations and seasonal conditions across both subsets. This validation strategy provides a defensible estimate of generalization for interpolation within observed conditions but does not fully characterize uncertainty associated with transfer beyond the sampled domain. Because stream systems often exhibit autocorrelation and regime-dependent behavior, alternative validation schemes that account for structured grouping (e.g., location- or season-based partitioning) would provide a more stringent test of extrapolative capacity and help mitigate the risk of optimistic performance estimates [25,30]. Likewise, comprehensive uncertainty quantification remains uncommon in the broader literature despite being central for decision support, and even pragmatic approaches such as bootstrap-based prediction intervals or Monte Carlo perturbations of inputs would strengthen interpretability for management applications [25,38]. The absence of explicit covariates such as suspended solids or turbidity, dissolved organic carbon fractions, and discharge implies that certain in-stream attenuation processes—including hyporheic exchange and dynamic partitioning—are captured indirectly through proxy predictors and co-metal covariance. This limitation is consistent with broader concerns that inadequate representation of attenuation and partitioning under dynamic conditions can bias predictions, regardless of whether models are mechanistic or empirical [7,12]. Future work that integrates direct hydrological measurements, higher-frequency event sampling, and hybrid modeling extensions (e.g., wavelet preprocessing, geographically weighted frameworks, or physically constrained ML) would improve transferability and further align predictions with source–transport–fate mechanisms under changing climatic or land-use conditions [23,24,27].

The collective evidence characterizes Ova Stream as a seasonally dynamic system in which trace metal distributions exhibit consistent spatial–temporal structuring within the observed environmental envelope. Rather than demonstrating direct mechanistic causality, the results indicate that predictability is element-specific and season-dependent, with patterns consistent with shifting influences of dilution processes, localized inputs, sediment interactions, and redox-related conditions. By integrating comparative regression performance, cross-season predictive evaluation, and interpretable feature-importance structures within a systematic monitoring framework, the study provides an empirically grounded template for seasonally resolved assessment of metal variability in urban streams. While the assembled dataset supports data-driven forecasting and screening applications, further incorporation of direct hydrological and sedimentary measurements would be necessary to fully resolve source–transport–fate mechanisms under evolving environmental conditions [10,11,14,25].

## 5. Conclusions

Trace metal behavior in the Ova Stream is organized primarily by seasonal hydrochemical reconfiguration rather than by any single dominant control. Dry-season conditions intensify concentration variability and localized enrichment—most notably for Cr and Cd—under reduced dilution and heightened sensitivity to point-source inputs and sediment–water coupling. In contrast, wet-season dynamics accentuate flow-driven connectivity and shifts in aqueous geochemical conditions, facilitating redistribution of metals along the stream continuum.

Ensemble ML models demonstrate consistently strong predictive skill, with RF and XGBoost outperforming alternative approaches. Feature-importance patterns resolve metal-specific control structures, in which temperature and dissolved oxygen act as integrative indicators of hydrological state and redox sensitivity, while nitrogen and phosphorus fractions, together with co-occurring metals, capture shared source signatures and coupled transport behavior.

These findings establish season-aware monitoring integrated with interpretable ML frameworks as a rigorous and operationally relevant approach for screening-level evaluation of trace metal contamination in urban stream systems, providing a data-driven complement to process-based analyses within environmental toxicology. Within this framework, season-aware and interpretable modeling offers a credible basis for resolving trace metal behavior under hydrologically dynamic conditions.

## Figures and Tables

**Table 1:** Statistical characterization of physicochemical conditions and trace metal signatures across at eight sampling points during the wet-season period.

Parameter	Minimum	Maximum	Mean	Standard Deviation	Skewness	Kurtosis
pH	7.49	8.87	7.96	0.42	0.90	−0.29
Temperature (°C)	11.40	13.20	12.22	0.54	0.36	−1.27
DO (mg L^−1^)	1.86	7.12	4.51	1.81	−0.12	−1.47
EC (µS cm^−1^)	33	104	56.39	21.07	0.92	−0.08
NH_3_ (mg L^−1^)	0.16	1.56	0.57	0.39	1.11	0.52
NO_2_^−^ (mg L^−1^)	0.03	0.23	0.10	0.06	0.58	−0.66
NO_3_^−^ (mg L^−1^)	0.58	5.77	1.65	1.52	1.90	2.37
TP (mg L^−1^)	0.24	4.48	0.64	0.67	3.82	14.59
TOP (mg L^−1^)	0.12	0.57	0.33	0.12	0.43	−0.38
SRP (mg L^−1^)	0.14	1.77	0.41	0.33	2.48	5.75
Cr (µg L^−1^)	0.03	0.81	0.18	0.19	2.01	3.22
Ni (µg L^−1^)	1.86	6.44	4.48	1.47	−0.24	−1.22
Cu (µg L^−1^)	0.001	6.20	1.29	2.03	1.41	0.47
As (µg L^−1^)	9.21	38.62	24.91	9.15	−0.33	−0.99
Cd (µg L^−1^)	0.001	0.09	0.008	0.02	3.05	8.14
Hg (µg L^−1^)	0.003	0.024	0.013	0.007	0.30	−1.16

**Table 2:** Statistical characterization of physicochemical conditions and trace metal signatures across at eight sampling points during the dry-season period.

Parameter	Minimum	Maximum	Mean	Standard Deviation	Skewness	Kurtosis
pH	5.90	8.88	7.65	0.74	−0.17	−0.77
Temperature (°C)	15.30	20.90	18.45	1.39	−0.62	−0.09
DO (mg L^−1^)	2.85	11.37	6.16	2.54	0.75	−0.81
EC (µS cm^−1^)	27	95	54.99	18.47	−0.04	−0.74
NH_3_ (mg L^−1^)	0.15	1.16	0.48	0.31	1.11	−0.26
NO_2_^−^ (mg L^−1^)	0.06	0.11	0.08	0.02	0.05	−1.26
NO_3_^−^ (mg L^−1^)	0.51	3.71	1.30	0.92	1.64	1.62
TP (mg L^−1^)	0.18	0.52	0.33	0.10	0.42	−0.97
TOP (mg L^−1^)	0.12	0.39	0.23	0.07	0.83	0.11
SRP (mg L^−1^)	0.10	0.32	0.19	0.06	0.50	−0.43
Cr (µg L^−1^)	0.03	22.23	3.21	6.18	2.50	4.76
Ni (µg L^−1^)	1.65	6.44	3.58	1.36	0.74	−0.32
Cu (µg L^−1^)	0.001	2.76	0.85	1.04	0.90	−0.94
As (µg L^−1^)	13.56	31.08	19.71	5.11	0.96	−0.08
Cd (µg L^−1^)	0.001	0.66	0.07	0.19	2.67	5.39
Hg (µg L^−1^)	0.011	0.038	0.02	0.009	1.12	−0.15

**Table 3:** Performance characteristics of leading regression models describing physicochemical regimes and nitrogen fraction dynamics.

Parameter	Best Algorithm	*R* ^2^	RMSE	MAE	MAPE
pH	RF	0.984	0.074	0.037	0.005
°C	XGBoost/RBF	0.998	0.158	0.081	0.005
DO	PR	0.993	0.174	0.121	0.027
EC	RF	0.990	2.012	1.017	0.019
NH_3_	RF	0.985	0.042	0.014	0.025
NO_2_^−^	RF	0.977	0.007	0.002	0.026
NO_3_^−^	XGBoost	0.994	0.100	0.039	0.028

**Table 4:** Performance characteristics of leading regression models describing phosphorus fractions dynamics.

Parameter	Best Algorithm	*R* ^2^	RMSE	MAE	MAPE
TP	RF	0.762	0.278	0.060	0.046
TOP	XGBoost Tree Ensemble	0.969	0.020	0.012	0.048
SRP	RF	0.903	0.070	0.049	0.057

**Table 5:** Performance comparison of regression models for Cr prediction. Best-performing metrics are shown in bold.

Model	*R* ^2^	MAE	RMSE	MAPE
**Random Forest Regression**	**0.997**	**0.105**	**0.314**	**0.759**
RBF Regressor	0.992	0.254	0.394	1.596
Polynomial Regression	0.976	0.497	0.686	4.733
XGBoost Tree Ensemble	0.968	0.203	0.910	0.154

**Table 6:** Performance comparison of regression models for Ni prediction. Best-performing metrics are shown in bold.

Model	*R* ^2^	MAE	RMSE	MAPE
**Random Forest Regression**	**0.992**	**0.065**	**0.127**	**0.019**
XGBoost Tree Ensemble	0.986	0.094	0.179	0.024
RBF Regressor	0.967	0.206	0.264	0.055
Polynomial Regression	0.969	0.185	0.253	0.051

**Table 7:** Performance comparison of regression models for Pb prediction. Best-performing metrics are shown in bold.

Model	*R* ^2^	MAE	RMSE	MAPE
**Random Forest Regression**	**0.945**	**0.007**	**0.011**	**0.041**
XGBoost Tree Ensemble	0.833	0.014	0.019	0.201
Polynomial Regression	0.816	0.018	0.021	0.174
RBF Regressor	0.784	0.019	0.024	0.325

**Table 8:** Performance comparison of regression models for Cd prediction. Best-performing metrics are shown in bold.

Model	*R* ^2^	MAE	RMSE	MAPE
**Random Forest Regression**	**0.992**	**0.004**	0.013	**0.827**
RBF Regressor	**0.992**	0.008	**0.011**	3.427
XGBoost Tree Ensemble	0.973	0.010	0.023	2.581
Polynomial Regression	0.968	0.017	0.024	9.647

**Table 9:** Performance comparison of regression models for As prediction. Best-performing metrics are shown in bold.

Model	*R* ^2^	MAE	RMSE	MAPE
**Random Forest Regression**	**0.995**	**0.244**	**0.582**	**0.013**
XGBoost Tree Ensemble	0.992	0.269	0.698	**0.013**
Polynomial Regression	0.983	0.588	1.001	0.034
RBF Regressor	0.973	1.017	1.322	0.053

**Table 10:** Performance comparison of regression models for Cu prediction. Best-performing metrics are shown in bold.

Model	*R* ^2^	MAE	RMSE	MAPE
**XGBoost Tree Ensemble**	**0.994**	0.054	0.128	3.042
Random Forest Regression	0.989	0.065	0.181	7.645
RBF Regressor	0.971	0.194	0.267	54.969
Polynomial Regression	0.965	0.240	0.344	57.459

**Table 11:** Performance comparison of regression models for Hg prediction. Best-performing metrics are shown in bold.

Model	*R* ^2^	MAE	RMSE	MAPE
**Random Forest Regression**	**0.985**	0.000	0.001	0.024
RBF Regressor	0.975	0.001	0.001	0.082
Polynomial Regression	0.962	0.001	0.002	0.087
XGBoost Tree Ensemble	0.751	0.003	0.005	0.434

**Table 12:** Gain-based ranking of the ten most influential predictors contributing to Cr model performance.

Rank	Feature	Gain	Weight	Cover	Total Gain	Total Cover
1	Temperature	181.2015	88	274.88	15,945.74	24,189
2	Hg	45.5627	17	152.65	774.57	2595
3	pH	12.8994	56	48.89	722.37	2738
4	As	5.2804	55	234.31	290.42	12,887
5	DO	2.8367	62	112.15	175.88	6953
6	NO_2_^−^	2.6311	30	88.47	78.93	2654
7	NO_3_^−^	1.3583	39	253.56	52.97	9889
8	EC	1.1119	21	168.10	23.35	3530
9	Pb	0.6612	23	119.91	15.21	2758
10	Cu	0.6254	35	226.97	21.89	7944

**Table 13:** Gain-based ranking of the ten most influential predictors contributing to Ni model performance.

Rank	Feature	Gain	Weight	Cover	Total Gain	Total Cover
1	NO_3_^−^	13.5289	73	216.78	987.61	15,825
2	DO	9.7716	27	96.41	263.83	2603
3	As	6.4202	36	106.92	231.13	3849
4	EC	3.2352	28	156.96	90.59	4395
5	Hg	1.6689	25	168.64	41.72	4216
6	NH_3_	0.7759	37	149.76	28.71	5541
7	Cd	0.7180	14	143.36	10.05	2007
8	TP	0.5260	33	156.15	17.36	5153
9	SRP	0.4948	44	185.73	21.77	8172
10	Cu	0.4270	32	139.22	13.66	4455

**Table 14:** Gain-based ranking of the ten most influential predictors contributing to Pb model performance.

Rank	Feature	Gain	Weight	Cover	Total Gain	Total Cover
1	Cr	0.00626	13	330.54	0.08132	4297
2	DO	0.00314	7	290.43	0.02198	2033
3	Cd	0.00282	5	206.20	0.01412	1031
4	NO_3_^−^	0.00260	13	243.85	0.03376	3170
5	Hg	0.00256	1	348.00	0.00256	348
6	NH_3_	0.00238	9	371.44	0.02146	3343
7	Cu	0.00206	13	368.77	0.02674	4794
8	Temperature	0.00203	18	179.44	0.03647	3230
9	As	0.00161	13	210.62	0.02095	2738
10	NO_2_^−^	0.00143	17	320.06	0.02429	5441

**Table 15:** Gain-based ranking of the ten most influential predictors contributing to Cd model performance.

Rank	Feature	Gain	Weight	Cover	Total Gain	Total Cover
1	NH_3_	0.20016	33	224.12	6.60517	7396
2	Temperature	0.15480	14	384.71	2.16715	5386
3	NO_3_^−^	0.01019	24	292.92	0.24455	7030
4	Pb	0.01028	13	333.08	0.13360	4330
5	pH	0.00649	13	76.54	0.08437	995
6	As	0.00380	14	243.57	0.05325	3410
7	TOP	0.00181	7	93.57	0.01266	655
8	NO_2_^−^	0.00164	12	462.58	0.01963	5551
9	Ni	0.00095	7	178.14	0.00667	1247
10	Cu	0.00095	13	171.00	0.01232	2223

**Table 16:** Comprehensive feature importance metrics for As prediction.

Rank	Feature	Weight	Gain	Cover	Total Gain	Total Cover
1	Hg	67	491.18	218.91	32,909.35	14,667
2	TOP	40	228.11	157.40	9124.41	6296
3	DO	39	58.04	79.97	2263.51	3119
4	TP	58	33.11	122.81	1920.56	7123
5	NO_3_^−^	80	19.13	135.40	1530.65	10,832
6	NO_2_^−^	73	8.81	131.40	643.20	9592
7	Ni	64	5.44	165.95	347.85	10,621
8	Cd	27	4.18	132.00	112.84	3564
9	Cr	68	3.98	91.65	270.83	6232
10	Pb	57	2.62	114.68	149.25	6537

## Data Availability

The datasets analyzed and generated during the current study are available from the corresponding author on reasonable request.

## Supplementary Materials

The following supporting information can be downloaded at: https://www.mdpi.com/article/10.3390/toxics14030215/s1, Figure S1: Point-based scatter plot showing the relationship between observed Cr concentrations and model-predicted Cr values. The distribution of points along the diagonal trend indicates the strength of the linear agreement between measured and predicted concentrations; Figure S2: Point-based scatter plot showing the relationship between observed Ni concentrations and model-predicted Ni values. The distribution of points along the diagonal trend indicates the strength of the linear agreement between measured and predicted concentrations; Figure S3: Point-based scatter plot showing the relationship between observed Cu concentrations and model-predicted Cu values. The distribution of points along the diagonal trend indicates the strength of the linear agreement between measured and predicted concentrations; Figure S4: Point-based scatter plot showing the relationship between observed As concentrations and model-predicted As values. The distribution of points along the diagonal trend indicates the strength of the linear agreement between measured and predicted concentrations; Figure S5: Point-based scatter plot showing the relationship between observed Cd concentrations and model-predicted Cd values. The distribution of points along the diagonal trend indicates the strength of the linear agreement between measured and predicted concentrations; Figure S6: Point-based scatter plot showing the relationship between observed Hg concentrations and model-predicted Hg values. The distribution of points along the diagonal trend indicates the strength of the linear agreement between measured and predicted concentrations; Figure S7: Point-based scatter plot showing the relationship between observed Pb concentrations and model-predicted Pb values. The distribution of points along the diagonal trend indicates the strength of the linear agreement between measured and predicted concentrations.

## Abbreviations

The following abbreviations are used in this manuscript:

ML	Machine Learning
RF	Random Forest
EC	Electrical Conductivity
DO	Dissolved Oxygen
NH_3_	Ammonia
NO_2_^−^	Nitrite
NO_3_^−^	Nitrate
TP	Total Phosphorus
TOP	Total Orthophosphate
SRP	Soluble Reactive Phosphorus
APHA	American Public Health Association
ICP-MS	Inductively Coupled Plasma-Mass Spectrometry
XGBoost	Extreme Gradient Boosting
RBF	Radial Basis Function
PR	Polynomial Regression
*R*^2^	The Coefficient of Determination
MAE	Mean Absolute Error
RMSE	Root Mean Squared Error
MAPE	Mean Absolute Percentage Error
Cr	Chromium
Cu	Copper
Ni	Nickel
Pb	Lead
Cd	Cadmium
Hg	Mercury
As	Arsenic
