# Supplementary material for: Environmental Drivers and Explainable Modeling to Resolve Trace Metal Dynamics in a Lotic System
Source: Toxics. 2026 Feb 28;14(3):215. doi: 10.3390/toxics14030215 (PMC13029825; doi:10.3390/toxics14030215)
Supplement: Supplementary file 1 [file toxics-14-00215-s001.zip › toxics-4157648-supplementary.pdf]

Supplementary File 1

The figures supporting model evaluation and interpretation

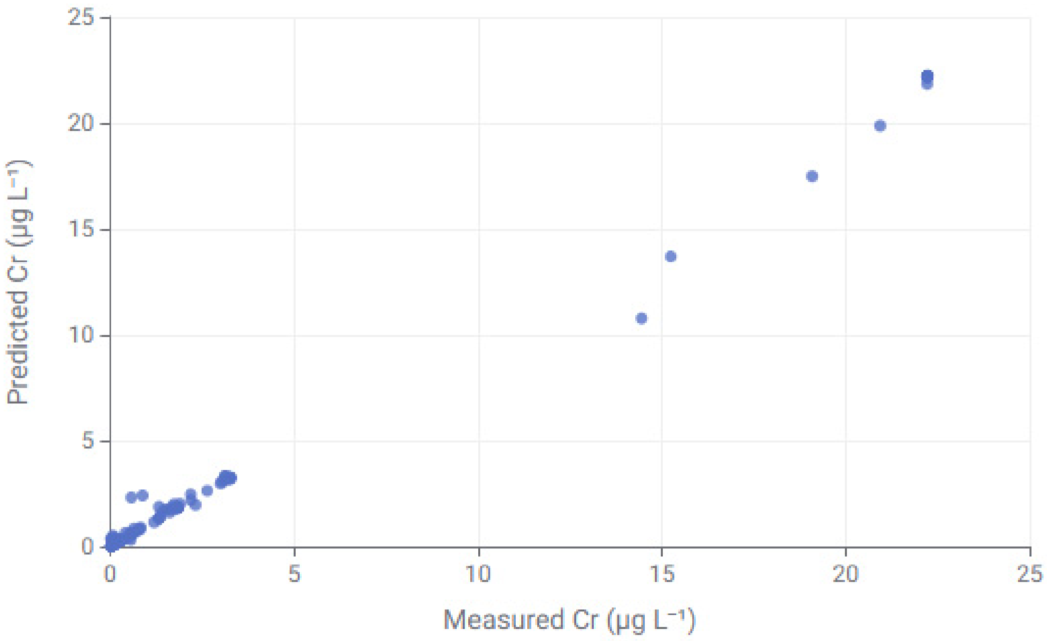

Figure S1. Point-based scatter plot showing the relationship between observed Cr concentrations and model-predicted Cr values. The distribution of points along the diagonal trend indicates the strength of the linear agreement between measured and predicted concentrations.

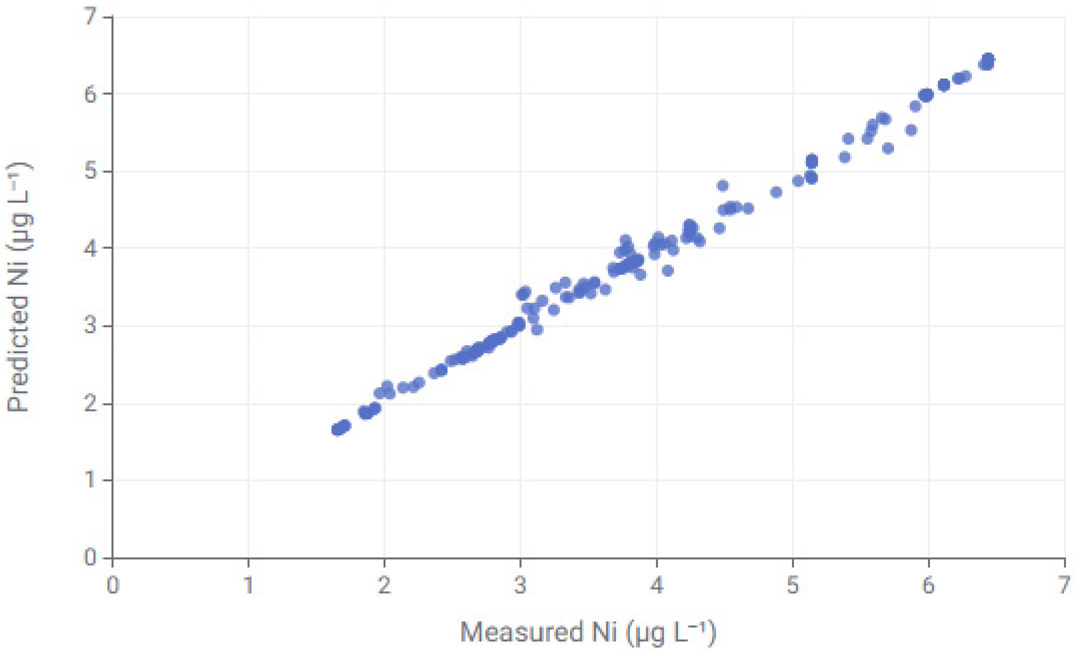

Figure S2. Point-based scatter plot showing the relationship between observed Ni concentrations and model-predicted Ni values. The distribution of points along the diagonal trend indicates the strength of the linear agreement between measured and predicted concentrations.

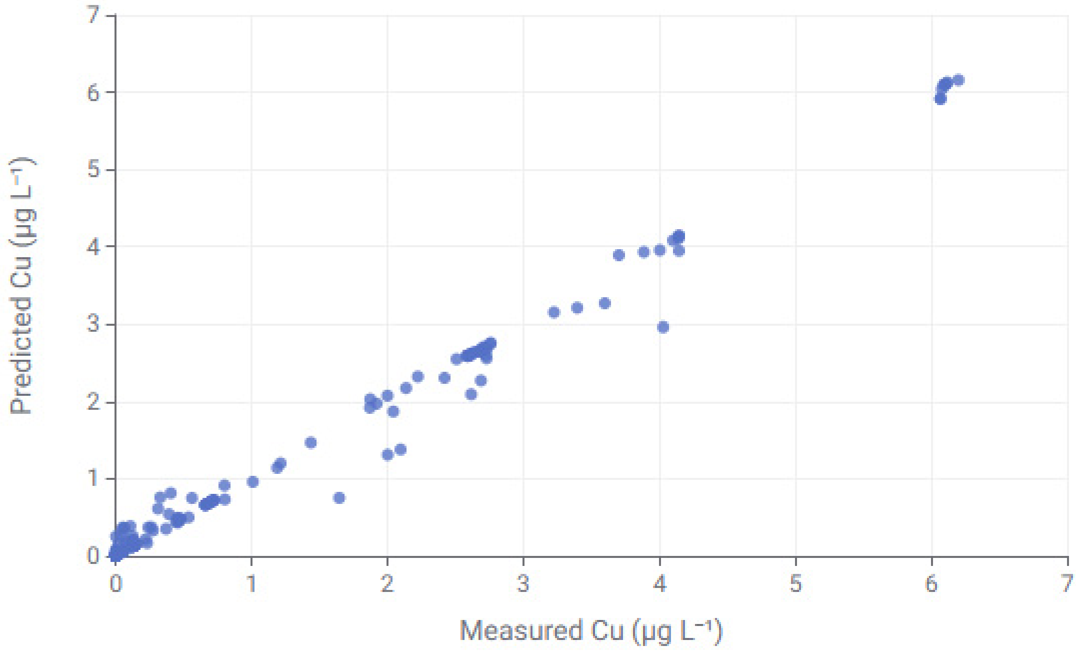

Figure S3. Point-based scatter plot showing the relationship between observed Cu concentrations and model-predicted Cu values. The distribution of points along the diagonal trend indicates the strength of the linear agreement between measured and predicted concentrations.

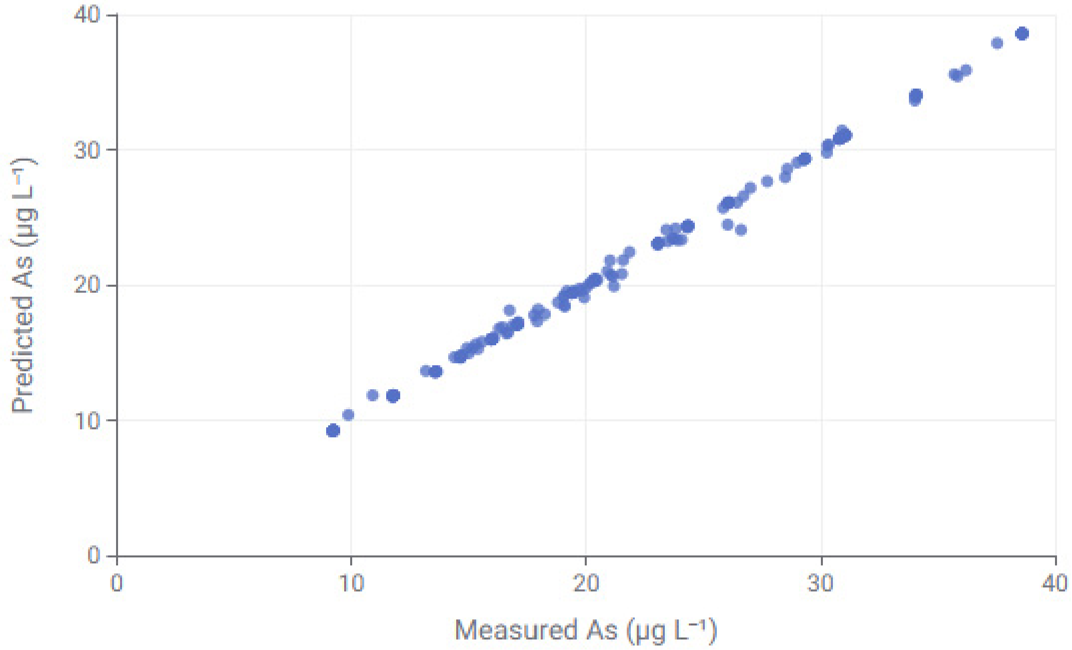

Figure S4. Point-based scatter plot showing the relationship between observed As concentrations and model-predicted As values. The distribution of points along the diagonal trend indicates the strength of the linear agreement between measured and predicted concentrations.

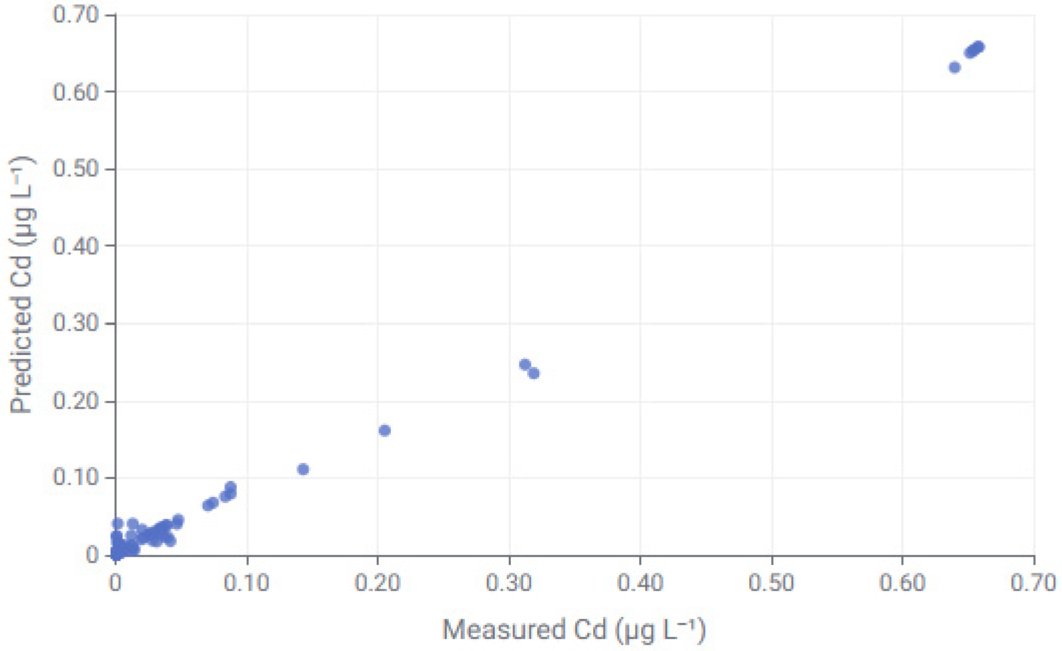

Figure S5. Point-based scatter plot showing the relationship between observed Cd concentrations and model-predicted Cd values. The distribution of points along the diagonal trend indicates the strength of the linear agreement between measured and predicted concentrations.

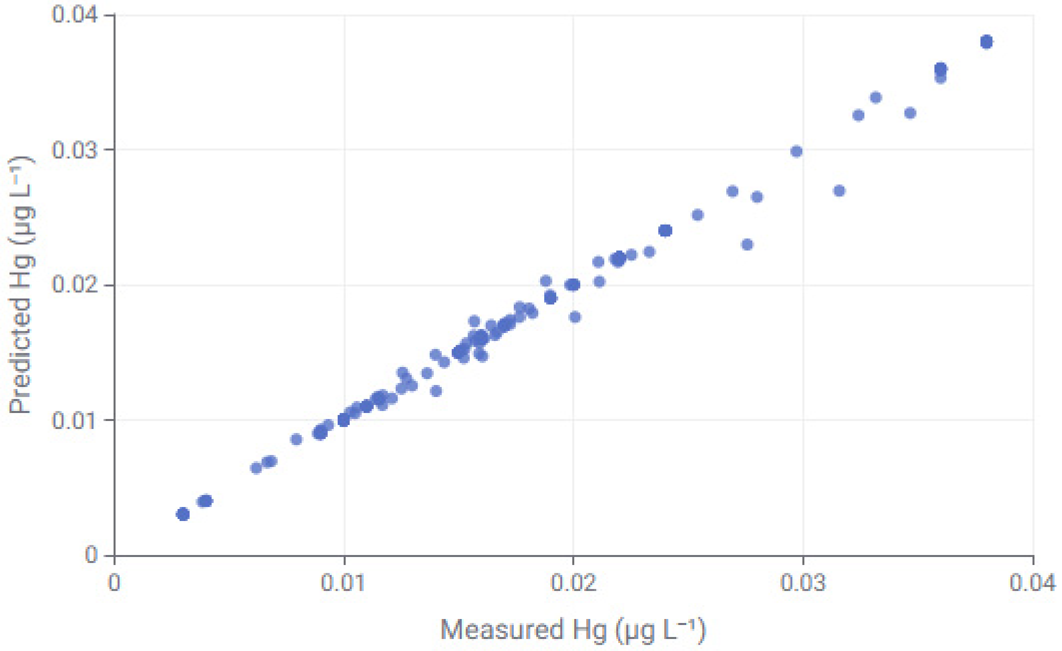

Figure S6. Point-based scatter plot showing the relationship between observed Hg concentrations and model-predicted Hg values. The distribution of points along the diagonal trend indicates the strength of the linear agreement between measured and predicted concentrations.

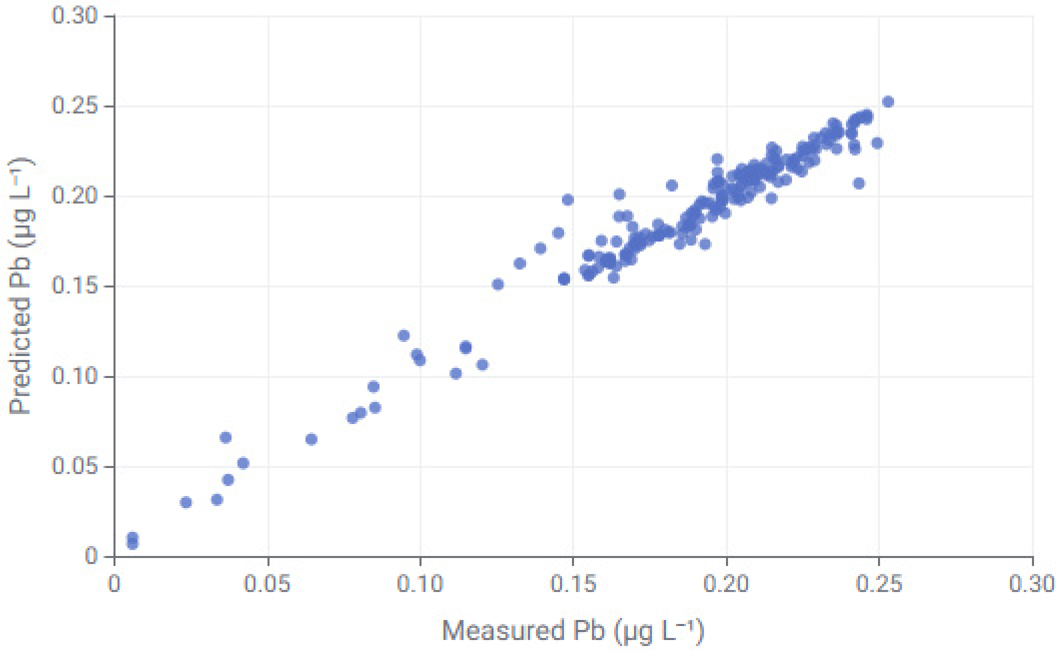

Figure S7. Point-based scatter plot showing the relationship between observed Pb concentrations and model-predicted Pb values. The distribution of points along the diagonal trend indicates the strength of the linear agreement between measured and predicted concentrations.
